# Carbolic Acid

**Published:** 1883-01

**Authors:** 


					﻿Carbolic acid, according to Dr. Robert J. Lee, is the only
antiseptic that can be volatilized in a definite and constant man-
ner. This is a most important fact in treatment, and deserving
attention. If a solution of one part of carbolic acid in eighty of
water be distilled under light pressure, the vapor will contain the
same proportion of the acid as the solution during the progress of
boiling; so that we can obtain vapor of any strength and diffuse
it in the atmosphere. Other antiseptics are often too volatile, as,
for example, thymol, which comes off very rapidly from the
boiling water, as does also benzonic acid, so that they are not con-
venient for inhaling.—Medical Record.
Proceedings.
The American Academy of Dental Science.
The fifteenth annual meeting of the American Academy of
Dental Science was held atYoung’s Hotel, Boston, October 25,1882.
The morning session was chiefly devoted to the regular business,
and to the election of the following-named officers: President, Dr.
G. T. Moffatt; vice president, Dr. J. H. Batcheller; recording
secretary, Dr. H. F. Hamilton ; corresponding secretary, Dr. E.
B. Hitchcock; treasurer, Dr. E. H. Smith; librarian, Dr. H. C.
Meriam; executive committee, Dr. C. P. Wilson, Dr. E. C.
Briggs and Dr. J. S. Mason. Dr. S. J. Shaw and Dr. F. E.
Banfield were elected active fellows. The afternoon session opened
with the annual address by Dr. Frank Abbot, of New York, on
“ Dental Education.” The paper was chiefly devoted to empha-
sizing the necessity of young men’s having a thorough medical ed-
				

